# 2335. Up-to-date COVID-19 vaccination coverage among healthcare personnel in nursing homes —National Healthcare Safety Network

**DOI:** 10.1093/ofid/ofad500.1957

**Published:** 2023-11-27

**Authors:** Sushmitha Ananth, Hannah E Reses, Heather Dubendris, Kira A Barbre, Emily Wong, Meng Lu, Jeneita Bell

**Affiliations:** Leidos, phoenix, Arizona; Centers for Disease Control and Prevention, Decatur, Georgia; CDC/Lantana, North Charleston, South Carolina; Goldbelt C6, Atlanta, Georgia; Centers for Disease Control and Prevention, Decatur, Georgia; Centers for Disease Control and Prevention, Decatur, Georgia; Centers for Disease Control and Prevention, Decatur, Georgia

## Abstract

**Background:**

To prevent morbidity and mortality associated with COVID-19 among nursing home residents, both residents and healthcare personnel (HCP) should remain up-to-date (UTD) with recommended COVID-19 vaccines, including the updated bivalent boosters. As of the first quarter of 2023 (December 26th, 2022 - March 26th, 2023) , the current Centers for Disease Control and Prevention (CDC) recommendation for UTD is defined as completion of primary series and receipt of bivalent booster, or primary series completion only if not yet eligible for booster. The objective of this project was to assess national COVID-19 UTD percentage among HCP in nursing homes by facility-characteristics.

**Methods:**

We analyzed vaccination data reported by nursing homes to the CDC’s National Healthcare Safety Network during January 23^rd^–February 5^th^, 2023. Based on the right-skewed distribution of the percentage of HCP who are UTD, median was selected as the main summary measure of central tendency. We calculated median UTD (%) nationally and by facility-size tertiles (measured by number of HCP that were eligible to have worked at a facility for at least 1 day during the week of data collection), social vulnerability index (SVI) tertiles, and urbanicity.

**Results:**

Among 14,857 nursing homes, median UTD (%) among HCP was 13.9% ; among smaller (< 92 total HCP) and larger facilities ( >145 total HCP) was 14.9% and 13.3%, respectively. Median UTD (%) for facilities located in low and high SVI areas was 15.3% and 12.8%, respectively. Median UTD (%) was 11.6% for facilities in non-metropolitan areas and 15.1% among those in metropolitan areas.

**Conclusion:**

COVID-19 median UTD (%) among HCP working in nursing homes in the U.S. was low overall and in all stratifications by facility size, SVI, and urbanicity. This suggests that additional strategies are needed to improve UTD coverage in this population.

**Disclosures:**

**All Authors**: No reported disclosures

